# Partner phubbing and its association with depression and family functioning: A cross-sectional study

**DOI:** 10.1097/MD.0000000000048104

**Published:** 2026-03-20

**Authors:** Dilan Onur, Zeliha Yelda Özer, Çağla Okyar, Servet Yüce

**Affiliations:** a Department of Family Medicine, Çukurova University Faculty of Medicine, Adana, Türkiye; b Department of Medical Education and Informatics, Çukurova University Faculty of Medicine, Adana, Türkiye; c Department of Public Health, İstanbul Provincial Health Directorate, İstanbul 34690, Türkiye.

**Keywords:** depression, digital neglect, family functioning, mental health, partner phubbing, PHQ-2, phubbing, primary care

## Abstract

The increasing integration of smartphones into daily life has given rise to partner phubbing – being ignored in favor of a partner phone use – which may impact mental health and family dynamics. Although phubbing has been explored in various relational contexts, empirical evidence on its association with depression and family functioning in primary care populations remains limited. This cross-sectional study was conducted among 222 married individuals aged 18 and above who visited a university-affiliated Family Health Center in Turkey between July and September 2024. Participants completed validated measures including the Phubbing Exposure Scale, Patient Health Questionnaire-2 and -9 (PHQ-2 and PHQ-9), and the Family Adaptability, Partnership, Growth, Affection, Resolve Scale. Statistical analyses included Mann–Whitney *U*, Kruskal–Wallis, chi-square, and correlation tests, with significance set at *P* < .05. Phubbing exposure was significantly higher among participants with positive PHQ-2 screenings for depressive symptoms (*P* < .01) and those with a history of psychiatric medication use (*P* < .01). Although PHQ-9 scores were not significantly associated with phubbing, early depressive symptoms measured by PHQ-2 were. Women reported higher phubbing exposure than men, and younger participants had higher perceived phubbing levels. Interestingly, individuals with low family functioning reported lower Phubbing Exposure Scale scores compared to those with moderate or high family functioning (*P* < .01), suggesting possible perceptual or relational dynamics in digitally neglected households. Partner phubbing appears to be linked with early depressive symptoms and various psychosocial characteristics, including gender, age, and psychiatric history. These findings highlight the relevance of digital neglect in clinical assessments and underscore the importance of integrating phubbing awareness into mental health and family care practices. Future research should explore longitudinal effects and develop interventions targeting smartphone-related disruptions in couple dynamics.

## 1. Introduction

With the rise of digital technology, smartphones have become one of the most common means of accessing the internet and social media platforms.^[1]^ While these tools offer unprecedented connectivity, they have also led to problematic usage patterns, including behavioral addictions.^[2]^ One such behavior – phubbing, 1st defined in 2012 by researchers at the University of Sydney – refers to the act of ignoring a person in a social setting by focusing on 1 smartphone instead.^[3]^ When this behavior occurs between romantic partners, it is termed partner phubbing**.^[4]^**

According to the World Health Organization, depression affects approximately 5% of adults globally, including 4% of men and 6% of women, with a prevalence of 5.7% among those aged 60 and older.^[5]^ Depression not only impairs individual well-being but can also disrupt interpersonal relationships and family dynamics.

Family functioning encompasses relational roles, emotional bonds, adaptability, and well-being. A healthy family system is 1 that adapts positively to changes and maintains effective communication and support mechanisms.^[6–8]^ Phubbing within intimate relationships has been associated with interpersonal conflict, reduced relationship satisfaction, and increased depressive symptoms.^[4]^ It may also impact family cohesion and disrupt healthy family functioning.

Despite the growing prevalence of phubbing behavior, empirical research exploring its relationship with mental health and family dynamics remains limited. Addressing this gap, the current study aimed to examine the association between exposure to partner phubbing, depression, and family functioning among married individuals presenting to a university-affiliated Family Health Center (EFHC) for any reason.

## 2. Methods

### 2.1. Ethical approval

The study protocol was approved by the Ethics Committee of Çukurova University Faculty of Medicine (Approval date: April 5, 2024; Decision No: 13).

### 2.2. Study design and population

This cross-sectional descriptive study was conducted among married individuals aged 18 years and older who were registered at the Çukurova University EFHC and presented to 1 of its 4 units for any reason during a 3-month period.

### 2.3. Sample selection

Participants were selected from married individuals who attended the Çukurova University EFHC between July 1 and September 30, 2024, and voluntarily agreed to participate. Participants were informed about the study through posters displayed in the waiting area and verbal communication provided by reception staff. Trained data collectors then conducted face-to-face interviews with those who expressed interest. Written informed consent was obtained from all participants.

Exclusion criteria included being unmarried, not using a smartphone, currently receiving medical treatment for depression, inability to provide informed consent, insufficient proficiency in the Turkish language, and the presence of acute physical or psychological conditions that could interfere with participation. The latter was determined at the time of eligibility screening by trained research personnel.

A sample size calculation was performed using G*Power 3.1 (Heinrich Heine University Düsseldorf, Düsseldorf, Germany). Based on an expected medium effect size (*f* = 0.25), 80% power, and an alpha level of 0.05 for 3-group comparisons (e.g., family functioning categories), the required sample size was calculated as 159. To account for potential nonresponse or incomplete data, the target sample size was increased by 25%, resulting in a final goal of approximately 200 participants.

Of the 265 individuals approached, 222 met the inclusion criteria and completed the study procedures. Twenty-seven individuals declined participation due to time constraints, and 16 were excluded due to acute illness or other medical concerns that were judged to impair participation reliability or safety.

One pregnant participant identified with depressive symptoms was referred to psychiatric services but did not receive pharmacological treatment. Follow-up care was provided to ensure her safety and well-being.

### 2.4. Data collection instruments

The following tools were used for data collection:

- **Sociodemographic Data Form:** Collected information on age, sex, marital status, economic status, presence of chronic or psychiatric illness, and psychiatric medication history.
- **Family Adaptability, Partnership, Growth, Affection, Resolve Scale:** Originally developed by Smilkstein and adapted into Turkish by Özcan et al, this 5-item tool assesses satisfaction with family functioning.^[9,10]^ Scores range from 0 to 10, with higher scores indicating greater satisfaction. Reliability coefficients (Cronbach alpha) range between 0.80 and 0.85.
- **Patient Health Questionnaire-2 (PHQ-2):** A 2-item screening tool assessing depressed mood and anhedonia over the past 2 weeks. A score of ≥ 3 indicates a positive screen and suggests possible major depressive disorder. It has a sensitivity of 97% and specificity of 67%.^[11]^
- **PHQ-9:** The PHQ-9 was administered to participants who screened positive on the PHQ-2 to assess the severity of depressive symptoms and support diagnostic confirmation. This 9-item self-report instrument evaluates the frequency of core depressive symptoms over the past 2 weeks, with total scores ranging from 0 to 27. Thresholds are commonly interpreted as follows: 0 to 4 (minimal), 5 to 9 (mild), 10 to 14 (moderate), 15 to 19 (moderately severe), and 20 to 27 (severe depression). The Turkish adaptation of the PHQ-9 was conducted by Sari et al,^[12]^ who reported strong diagnostic validity and internal consistency for use in primary care populations. The Cronbach alpha coefficient for the 9 items was 0.842, indicating high reliability.
- **Phubbing Exposure Scale (PES):** Developed by Chotpitayasunondh and Douglas^[13]^ and adapted to Turkish by Ergün et al^[14]^. This 22-item, 7-point Likert scale assesses partner phubbing across 3 subscales: Perceived Norms (PN), Feeling Ignored, and Interpersonal Conflict (IC). Internal consistency (Cronbach alpha) ranges from 0.92 to 0.97.

### 2.5. Data collection procedure

All instruments were administered via face-to-face interviews by trained personnel. Data collection was carried out between July 1 and September 30, 2024. Participation was voluntary, and informed consent was obtained from all respondents.

### 2.6. Statistical snalysis

Categorical variables were summarized as frequencies and percentages, while continuous variables were presented as mean ± standard deviation or, when appropriate, as median with minimum and maximum values. The chi-square test was used to compare categorical variables between groups. The Kolmogorov–Smirnov test assessed the normality of continuous variables. For nonnormally distributed data, the Mann–Whitney *U* test was used to compare 2 groups, and the Kruskal–Wallis test was applied for comparisons involving more than 2 groups. Post hoc pairwise comparisons were performed using the Bonferroni-adjusted Mann–Whitney *U* test. Pearson or Spearman correlation analyses were conducted to examine associations between continuous variables, depending on distributional assumptions. All statistical analyses were performed using IBM Statistical Package for the Social Sciences Statistics version 20.0 (IBM Corp., Armonk), with a significance level set at *P* < .05.

## 3. Results

A total of 222 married individuals participated in this cross-sectional study, with a mean age of 34.5 ± 8.4 years (range: 23–60); 69.4% of participants were female. Most were currently employed (70.2%) and held at least a university degree (68.9%). The largest proportion of participants (45.5%) reported a monthly household income above 50,001 TL (approximately 1500 USD). Chronic diseases were reported by 17.1% and psychiatric conditions by 7.7%, while 23.4% had a history of psychiatric medication use (Table 1).

**Table 1:** Sociodemographic characteristics of participants.

Variable	Value
Age (yr), mean ± SD	34.5 ± 8.4
Gender, n (%)	
Female	154 (69.4%)
Male	68 (30.6%)
Employment status, n (%)	
Employed	156 (70.2%)
Unemployed	66 (29.8%)
Education level, n (%)	
University or higher	153 (68.9%)
High school or below	69 (31.1%)
Monthly income, n (%)	
>50,001 TL (>1500 USD)	101 (45.5%)
≤50,000 TL (≤1500 USD)	121 (54.5%)
Chronic disease present, n (%)	38 (17.1%)
Previous psychiatric diagnosis, n (%)	17 (7.7%)
History of psychiatric medication, n (%)	52 (23.4%)

SD = standard deviation.

**Figure 1.**
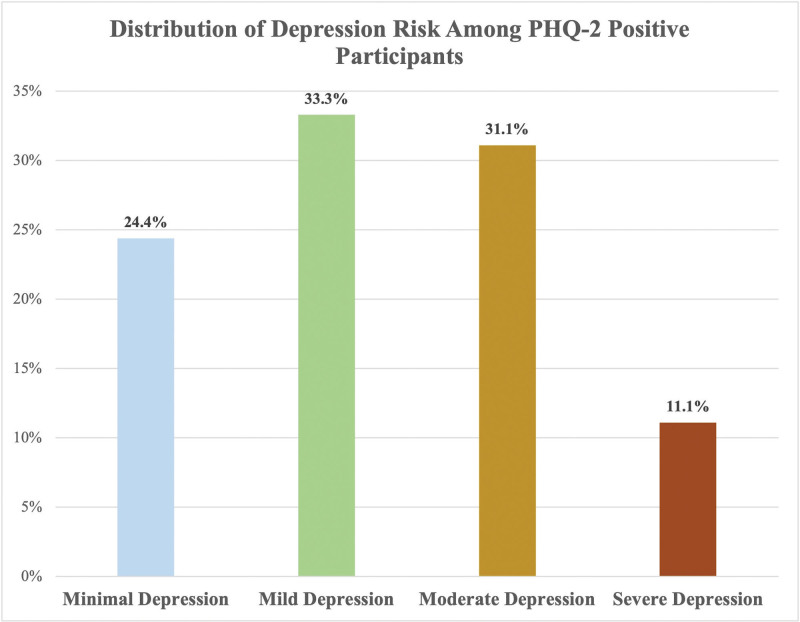
Bar chart showing the distribution of depression risk among PHQ-2 positive participants based on PHQ-9 categories. PHQ = Patient Health Questionnaire.

Based on PHQ-2 screening, 45 participants (20.3%) scored ≥ 3, indicating a positive screen for depressive symptoms. Among PHQ-2–positive individuals, PHQ-9 assessments revealed that 33.3% were at risk for mild, 31.1% for moderate, 24.4% for minimal, and 11.1% for severe depression (Fig. 1). No statistically significant associations were found between PHQ-9 scores and PES subscales (*P* > .05).

These individuals had significantly higher median scores on all 3 subscales of the PES: PN, Feeling Ignored, and IC (*P* = .001, *P* = .006, and *P* = .001, respectively) (Table 2). A negative correlation was observed between PHQ-2 scores and age (*P* = .026).

**Table 2:** PHQ-2 screening and Phubbing Exposure Scores.

Group	Perceived Norms (PN), median	Feeling Ignored (FI), median	Interpersonal Conflict (IC), median
PHQ-2 positive (n = 45)	36 (12–59)	22 (8–55)	9 (5–35)
PHQ-2 negative (n = 177)	25 (9–63)	16 (8–56)	13 (5–34)
*P*-value	.001*	.006*	.001*

Mann–Whitney *U* test was used.

PHQ = Patient Health Questionnaire.

* Indicates significant difference.

**Figure 2.**
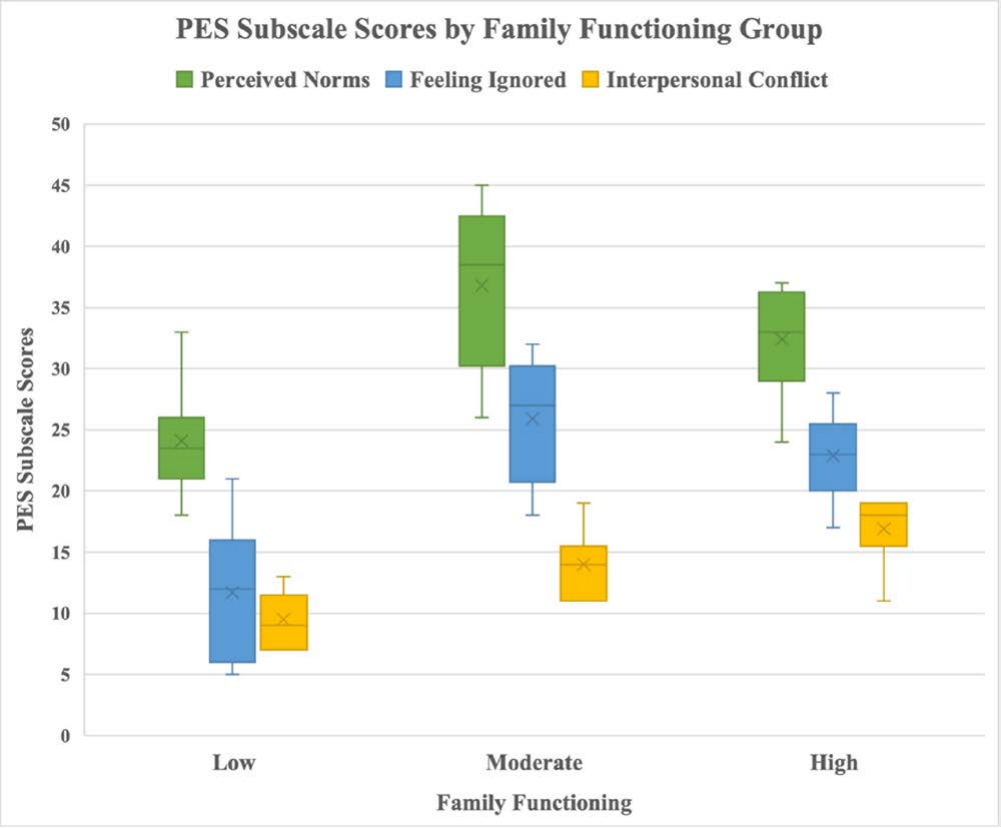
Boxplot comparing Phubbing Exposure Scale (PES) subscale scores (perceived norms, feeling ignored, and interpersonal conflict) across different levels of family functioning (low, moderate, and high).

In terms of family functioning, 74.8% of participants were categorized as having high function according to the Family APGAR Scale, while 20.7% had moderate and 4.5% had low function. PES scores differed significantly across APGAR groups (*P* < .01), with participants in the low-functioning group reporting the lowest levels of perceived phubbing (Table 3 and Fig. 2).

**Table 3:** Family functioning and PES scores.

Family APGAR group	Perceived Norms (PN), median	Feeling Ignored (FI), median	Interpersonal Conflict (IC), median
Low	25 (9–61)*	15 (8–56)*	9 (5–35)*
Moderate	35 (13–59)	25 (8–56)	15 (5–34)
High	32 (15–63)	22 (8–56)	14 (5–35)
*P* value	.006	.005	.009

Kruskal–Wallis test was used.

APGAR = Adaptability, Partnership, Growth, Affection, Resolve, PES = Phubbing Exposure Scale.

* Indicates the most significant different group.

Gender differences were significant: women scored higher than men across all PES subscales (*P* = .014, *P* = .001, *P* = .005). A weak negative correlation was observed between age and both PN and IC scores (*r* = −0.142 and *r* = −0.138, respectively).

Participants without chronic illness had significantly higher PN scores (*P* = .040), while those with psychiatric illness reported significantly higher IC scores (*P* = .030). Additionally, participants with a history of psychiatric medication use had significantly higher scores across all PES subscales (*P* = .013, *P* = .001, *P* = .001).

No significant differences were observed in PES scores based on education level, occupation, family type, or household income (*P* > .05).

## 4. Discussion

This study was designed to examine the relationship between exposure to partner phubbing, depressive symptoms, and family functioning among married individuals attending a university-affiliated primary care center. The main findings indicate that partner phubbing is significantly associated with both depressive symptoms and levels of family functioning.

Although the concept of phubbing has been increasingly explored in recent years, the literature especially regarding partner phubbing, remains limited. Among the existing studies, gender differences are frequently examined.^[15]^ Consistent with prior findings, our study revealed that women reported significantly higher levels of phubbing exposure compared to men. This may reflect the influence of gender roles and emotional expectations on the perception of digital neglect.^[16]^ It is plausible that phubbing behavior exerts a more profound emotional impact on women.^[17]^ However, the predominance of women in our sample may have also influenced this gender-related finding.

Regarding age, our study identified a weak negative correlation between age and levels of phubbing exposure. This suggests that as age increases, perceived exposure to partner phubbing tends to decrease. One plausible explanation is that younger individuals are more deeply integrated with digital technologies, making them more aware of – or more sensitive to – digital neglect within interpersonal relationships. Similar findings have been reported in the literature.^[18]^ However, contrasting studies have also indicated that partner phubbing levels do not significantly vary with age, highlighting some inconsistency in existing evidence and the need for further research on age-related differences in phubbing perception.^[17]^

In our analysis of chronic health conditions, only the PN subscale showed a statistically significant association. Specifically, individuals without chronic illness reported higher PN scores than those with chronic conditions. This could be explained by the fact that chronic diseases are more prevalent in older adults, whose health concerns may overshadow their sensitivity to partner phubbing, making them less likely to perceive such behavior as problematic.

Notably, the presence of a psychiatric disorder was significantly associated with higher scores only on the IC subscale. Individuals with a psychiatric history may interpret their partner phone use as intentional neglect, potentially intensifying relational conflict. Since the IC subscale reflects more overt and tangible relationship disturbances, these issues may be more readily perceived by individuals with existing mental health vulnerabilities. In contrast, the more abstract dimensions of phubbing – such as perceived norms or feelings of being ignored – may not be as easily identified or linked to psychiatric history.

Participants with a history of psychiatric medication use reported significantly higher levels of partner phubbing exposure. This may reflect heightened emotional sensitivity among these individuals, making them more acutely aware of or more emotionally impacted by perceived digital neglect. They may also be more inclined to interpret their partner preoccupation with their phone as a form of personal rejection or emotional disengagement.

Our findings indicate a significant increase in PHQ-2 scores, a tool designed to screen for early depressive symptoms, with escalating exposure to phubbing. However, no statistically significant association was found between phubbing exposure and PHQ-9 scores, which assess the broader severity and complexity of depressive symptoms, including somatic and cognitive manifestations. This pattern suggests that phubbing may be more directly linked to the initial emotional indicators of depression, such as anhedonia and low mood, rather than the more complex array of symptoms captured by the PHQ-9. The PHQ-2 role as a screening instrument for depression risk, rather than a diagnostic tool, may explain its greater sensitivity in capturing the psychological impact of partner phubbing compared to the PHQ-9. This finding suggests that partner phubbing could be an early relationship stressor contributing to the initial stages of depressive symptom development, potentially serving as a warning sign before more severe depression emerges. From a clinical perspective, these findings indicate that primary care providers should consider partner phubbing as a potential early indicator of relationship issues and nascent depressive symptoms. The association between phubbing exposure and depression screening results, rather than established depression severity, suggests that intervention at this stage could be particularly valuable for prevention. Primary care providers could incorporate brief assessments of digital behaviors and their impact on intimate relationships as part of routine mental health screenings, thereby identifying at-risk individuals before depressive symptoms become more severe. In this context, including phubbing awareness and digital neglect in family medicine residency training is critically important.

On the other hand, the relationship between phubbing and early depressive symptoms should not be assumed to imply causality. While phubbing exposure may trigger emotional distress in individuals, it is also possible that emotionally vulnerable individuals are more susceptible to their partner digital disengagement. The inherently bidirectional nature of such relationships is difficult to explain with data from cross-sectional studies. The differential association between PHQ-2 and PHQ-9 underscores the importance of distinguishing between early symptom detection and the severity of clinical depression in future research designs. Therefore, longitudinal and experimental studies are essential to gain a clearer understanding of the direction of this relationship and the underlying psychosocial mechanisms.

The finding that PHQ-2–positive individuals had significantly higher scores across all subscales of the PES reinforces the notion that partner phubbing may have a meaningful impact on mental health. This aligns with previous studies, which have also reported a negative relationship between phubbing and psychological well-being.^[19–21]^

An especially noteworthy finding concerns family functioning. Participants categorized as having low family functioning reported lower levels of perceived phubbing. While this may appear paradoxical, it is possible that in families with poor communication, phubbing behaviors are less likely to be noticed or perceived as problematic. Alternatively, intense phubbing may have already contributed to the erosion of family cohesion and communication, making such behaviors feel normalized or less conspicuous.^[22]^

### 4.1. Study limitations

This study has several limitations. Due to its cross-sectional design, causal relationships cannot be established – only associations can be inferred. The sample was limited to individuals presenting to a university-affiliated family health center, which may limit the generalizability of findings to the broader population. Additionally, data were based on self-report measures, introducing potential bias. Moreover, phubbing behavior was assessed only from the participant perception; the partner actual behavior was not evaluated objectively.

### 4.2. Conclusion and recommendations

The findings of this study highlight the potential impact of partner phubbing on the mental health and family dynamics of married individuals. Recognizing partner phubbing as a modern communication problem driven by contemporary lifestyles, and addressing its effects in clinical settings, may help broaden the perspective of family medicine practice. As such, incorporating the concept of digital neglect and phubbing into both clinical evaluations and educational programs is of critical importance for supporting the health of individuals and families.

Future research should employ longitudinal designs to explore the long-term effects of phubbing on family functioning and utilize multicenter data collection to enable more in-depth analysis. Furthermore, the development of educational and intervention programs aimed at reducing phubbing behavior is warranted. Medical students and resident physicians should be equipped with the skills to evaluate the negative consequences of digital technology use at both individual and family levels. They should also be encouraged to inquire about phubbing during mental health screenings. Additionally, integrating communication-focused counseling techniques and mindfulness-based approaches into training curricula may enhance the quality of care delivered by family physicians in real-world practice.

## Acknowledgments

We would like to thank the staff of the Çukurova University Educational Family Health Center for their support during the data collection process, and all participants who voluntarily contributed to this study.

## Author contributions

**Conceptualization:** Dilan Onur, Zeliha Yelda Özer.

**Data curation:** Dilan Onur, Zeliha Yelda Özer, Çağla Okyar.

**Formal analysis:** Dilan Onur, Zeliha Yelda Özer, Servet Yüce.

**Funding acquisition:** Dilan Onur, Zeliha Yelda Özer.

**Investigation:** Dilan Onur, Zeliha Yelda Özer, Çağla Okyar, Servet Yüce.

**Methodology:** Dilan Onur, Zeliha Yelda Özer, Çağla Okyar, Servet Yüce.

**Project administration:** Dilan Onur, Zeliha Yelda Özer, Servet Yüce.

**Resources:** Dilan Onur, Zeliha Yelda Özer, Çağla Okyar.

**Software:** Dilan Onur, Zeliha Yelda Özer.

**Supervision:** Dilan Onur, Zeliha Yelda Özer, Çağla Okyar, Servet Yüce.

**Validation:** Dilan Onur, Zeliha Yelda Özer, Çağla Okyar, Servet Yüce.

**Visualization:** Dilan Onur, Zeliha Yelda Özer, Çağla Okyar, Servet Yüce.

**Writing – original draft:** Dilan Onur, Zeliha Yelda Özer, Çağla Okyar, Servet Yüce.

**Writing – review & editing:** Dilan Onur, Zeliha Yelda Özer, Çağla Okyar, Servet Yüce.
